# Integrating Soluble Biomarkers and Imaging Technologies in the Identification of Vulnerable Atherosclerotic Patients

**Published:** 2007-02-07

**Authors:** José A. Páramo, José A. Rodríguez JA, Josune Orbe

**Affiliations:** Atherosclerosis Research, Division of Cardiovascular Sciences, CIMA, University of Navarra, Pamplona, Spain

**Keywords:** Biomarkers, Atherosclerosis, Inflammation, Vulnerable plaque, Proteolysis, Metalloproteinases

## Abstract

The clinical utility of a biomarker depends on its ability to identify high-risk individuals to optimally manage the patient. A new biomarker would be of clinical value if it is accurate and reliable, provides good sensitivity and specificity, and is available for widespread application. Data are accumulating on the potential clinical utility of integrating imaging technologies and circulating biomarkers for the identification of vulnerable (high-risk) cardiovascular patients. A multi-biomarker strategy consisting of markers of inflammation, hemostasis and thrombosis, proteolysis and oxidative stress, combined with new imaging modalities (optical coherence tomography, virtual histology plus IVUS, PET) can increase our ability to identify such thombosis-prone patients. In an ideal scenario, cardiovascular biomarkers and imaging combined will provide a better diagnostic tool to identify high-risk individuals and also more efficient methods for effective therapies to reduce such cardiovascular risk. However, additional studies are required in order to show that this approach can contribute to improved diagnostic and therapeutic of atherosclerotic disease.

## Introduction

Atherosclerotic disease and its thrombotic complications (atherothrombosis) remain the leading cause of mortality and morbidity in Western society. The mortality associated with atherosclerotic disease is related to the acute coronary syndromes (ACS), including acute myocardial infarction, unstable angina pectoris and sudden cardiac death. There is substantial clinical, experimental and postmortem evidence demonstrating the role of acute thrombosis on disrupted atherosclerotic plaques in the onset of ACS. Atherosclerotic plaque composition, rather than stenotic severity, appears to be central in determining risk of both plaque rupture and subsequent thrombogenicity. Inflammation plays a central role throughout the entire disease progression, and it lies at the root of atherosclerosis and its complications (1–3). Plaques within the coronary circulation become “high-risk” (vulnerable plaque) in response to a wide array of local and systemic influences/atherosclerotic risk factors. Thrombus formation in association with these lesions may be accelerated or amplified under the same influences (vulnerable blood). Similarly, at risk myocardium that is prone to rhythm disturbances or subject to ischemic flow is likely to experience dysfunction (vulnerable myocardium). Therefore, a combination of risk factors contributes to a vulnerable plaque composition, prothrombotic milieu, and susceptible heart, conditions that strongly favor the clinical manifestations of ACS (4). We are currently limited in our ability to identify accurately patients at risk of ACS. However, new imaging modalities in combination with the development of new biomarkers (bioimaging) may improve our understanding and management of patients at risk of coronary artery disease in the new millennium.

## Atherosclerotic Plaque Imaging

There is growing evidence that different types of vulnerable plaques exist that have different functional and biological features. Plaques may have similar structural features and morphologic assessment, but may differ in their biology, their activity, and thus their likelihood of advancing toward clinical complications.

In the past, invasive coronary angiography has been the only diagnostic procedure for identifying coronary atherosclerosis. Angiography provides purely morphologic information about vessel lumen diameter. However, various clinical observations in recent years have emphasized the need for more detailed analysis of atherosclerotic plaques. Several methods are available that provide detailed information about vessel wall and plaque morphology (Table 1). The goal of these techniques is to identify vascular remodeling and describe plaque with regard to specific morphologic criteria concerning vulnerability (5).

Intravascular ultrasound (IVUS) is a catheter-based technology that allows for assessment of vessel wall thickness and structure. Coronary angioscopy also allows to visualize the vessel lumen. But, because of their invasive nature, these techniques are not suitable as screening methods. More recently, optical coherence tomography (OCT) and virtual histology/IVUS have been introduced as other invasive techniques that provide images of vessel wall morphology and plaque characteristics. In contrast to these invasive approaches, noninvasive technologies such as electron beam computed tomography (EBCT) allow for high resolution assessment of coronary artery luminal morphology. This method is characterized by a high negative predictive value for exclusion of coronary disease (6–9). Some studies also suggest a potential value for determination of plaque composition and vulnerability (10). Finally, a variety of approaches at the molecular level (e.g. positron emission tomography, Technetium99m-labeled annexin V), targeting plaque inflammation, apoptosis, smooth muscle cell proliferation, extracellular matrix (ECM) activation, or platelet binding, have been recently introduced (11).

## Biomarkers of “vulnerable blood”

Biomarkers are generally considered to be systemic measurements of molecules, proteins, or enzymes that provide independent diagnostic or prognostic value by reflecting an underlying disease state or condition. The clinical utility of a biomarker depends on its ability to identify high-risk patients, be accurate and reliable, and provide good sensitivity, specificity and predictive value. Clinical application further requires the demonstration that evaluation of the biomarker is not only predictive of disease, but also adds predictive value to traditional risk factors and global vascular risk assessment, such as the Framingham score. In the case of coronary artery disease, the marker must reflect the underlying biology of the vessel wall, quantifying cardiovascular specific inflammation, thereby predicting the risk of recurrent atherothrombosis and its sequelae (12, 13). Historically, cholesterol and, in particular, LDL-cholesterol, has been considered the prototypical risk factor for coronary artery disease. However, lipoproteins alone do not explain all the coronary artery disease risk; one-half of all heart attacks and strokes occur among individuals without hypercholesterolemia, and one-fifth of all cardiovascular events occur in the absence of any of the major risk factors. Additional and new biomarkers are therefore needed to diagnose and prognosticate coronary artery disease more precisely (Table 2) (14).

### Cytokines

Cytokines are pleiotropic proteins that regulate leukocyte activity. Interleukin-6, monocyte chemoattractant protein-1(MCP-1) and TNF-α have shown promise in the prediction of coronary heart disease (15). Patients with ACS have increased circulating levels of IL-6 compared with those patients with stable angina (16). Measurement of MCP-1 in the coronary sinus blood of patients with unstable angina demonstrate an association with the extent of coronary atherosclerosis as assessed by angiography (17). In the CARE trial in patients with a recent myocardial infarction (MI), those who experienced a recurrent MI or cardiac death had higher TNF-α levels than matched controls (18). However, as the number of cytokines implicated in ACS increases, it will become important to determine whether they provide independent prognostic information apart from more established inflammatory biomarkers such as C-reactive protein.

### C-Reactive protein (CRP) and other acute-phase reactants

Elevated CRP has been associated with undiagnosed peripheral, coronary, and cerebral artery disease; it differentiates patients with unstable versus stable angina; predicts future MI, stroke and sudden cardiac death in patients with coronary artery disease; correlates with MI; and predicts the presence, degree, and symptomatology of carotid stenosis, as well as early morbidity and late mortality following coronary artery bypass grafting, and late restenosis following percutaneous cardiac interventions. A major advantage of CRP over the other inflammatory biomarkers is evidence that independently adds predictive power to both lipid screening and the Framingham risk score (19–21). Furthermore, a precise standardised, commercially available assay designed for cardiovascular risk assessment is widely available, complete with accepted normal ranges and screening guidelines from the American Heart Association and the Center for Disease Control (22). Beyond CRP’s ability to predict risk for both primary and secondary prevention of cardiovascular disease, interest has increased with the recognition that statins lower CRP in a manner largely independent of LDL-C reduction (23, 24).

In addition to its function as a component of the generalized hepatic response to acute inflammation, CRP also appears to be produced locally in atherosclerotic plaques by resident macrophages and vascular smooth muscle cells, and may be involved in several important steps in plaque genesis, progression and rupture. CRP upregulates endothelial cell permeability, promotes endothelial adhesion, is present in subintimal plaque, fixes complement, recruits monocytes and macrophages to foci of endovascular inflammation, and stimulates local thrombogenesis. CRP may also be involved in foam cell generation, as it binds oxidized LDL with high affinity, and the resultant CRP-LDL aggregates are taken up by macrophages (25, 26). A novel finding recently reported by our group is that CRP induces endothelial expression of metalloproteinases (MMPs) capable of degrading the extracellular matrix. CRP increased the expression of MMP-1 (collagenase) and MMP-10 (stromelysin-2) by human endothelial cells via kinase pathways. In addition, subjects with CRP values >3mg/L had increased plasma MMP-1 and –10. Finally, CRP and MMP-10 colocalized within the endothelial layer and macrophage-rich areas in advanced atherosclerotic plaques. Our results show increased local and systemic CRP-related MMP activation, thus providing a link between inflammation and plaque vulnerability (27). These findings suggest that CRP may directly mediate vascular tissue injury through pro-inflammatory, pro-thrombotic and proteolytic actions.

Fibrinogen may increase cardiovascular risk because of its role in fibrin formation, platelet aggregation and plasma viscosity, and is also an acute-phase reactant that is elevated in inflammatory states. Levels of fibrinogen may also be useful in the identification of cardiovascular risk patients (28). The role of other acute-phase reactants, such as serum amyloid A, in the prediction of coronary risk has not been established.

### Biomarkers of endothelial cell activation

Plasma derived soluble forms of the immunoglobulin family members intercellular adhesion molecule-1 (sICAM-1) and vascular cell adhesion molecule-1 (sVCAM-1) have been examined as possible biomarkers in ACS (29). On the basis of studies in the acute setting it does not appear that sICAM is useful in risk stratification of patients with ACS (30). In a prospective study of patients with non-ST elevation MI (NSTEMI), sVCAM were significantly higher at presentation in patients who had a major adverse cardiovascular events at 6 months. However, more prospective studies are required before sVCAM can be validated as a marker of coronary risk. The major utility of E-selectin and other markers of endothelial cell activation may be in predicting risk of developing coronary artery disease in patients with stable lesions (31). In patients with ACS, von Willebrand factor (vWF) levels are raised at admission, which may reflect endothelial dysfunction/damage. vWF is an independent predictor of adverse outcomes in ACS, and also a biomarker of subclinical atherosclerosis, although clinical implications for an individual patient remain unclear (32–34).

### Markers of oxidative stress

Oxidative stress leading to modification of LDL is an important mechanism of atherogenesis and plaque destabilization. Recent results suggest that markers of oxidative stress may have prognostic significance in ACS (35).

Myeloperoxidase (MPO) is produced by neutrophils and monocytes at sites of inflammation. MPO can generate several reactive, oxidatively modified intermediates able to induce oxidative damage to cells and tissues. Accumulating evidence suggests that MPO may play a role in plaque vulnerability. In a prospective study, patients with ACS and elevated MPO levels had a statistically significant increase in the risk of death or MI during the first 72 hours, independently of troponin or CRP, suggesting that MPO provides independent prognostic information distinct from other established biomarkers. Advanced human atherosclerotic plaques from patients with sudden cardiac death, strongly expressed MPO at sites of rupture. The most important use of MPO may be early risk stratification of patients with NSTEMI (36).

Oxidized-LDL (Ox-LDL) is generated during lipid peroxidation, resulting in generation of reactive species that modify the lipid components of LDL. Ox-LDL leads to foam cell formation and elaboration of pro-inflammatory cytokines that promote endothelial dysfunction. Currently three major Ox-LDL plasma ELISAs based on murine monoclonal antibodies have been developed. Circulating levels of Ox-LDL have been associated with the presence of coronary artery disease in patients undergoing elective angiography; similarly elevated Ox-LDL levels are significantly higher in patients with ACS, finally, some but not all prospective studies have shown higher Ox-LDL levels in patients subsequent development of cardiac death, non-fatal MI, and unstable angina. Therefore, Ox-LDL is an attractive biomarker, as it may provide a link between lipoprotein disorders and inflammation (37, 38).

Lipoprotein-associated phospholipase A_2_ (Lp-PLA_2_) is a calcium-independent serine lipase that is associated with LDL in human plasma and serum. It is produced by macrophages and its expression is increased in atherosclerotic lesions (39, 40). A recent analysis of patients from the GRACE study found that elevated LP-PLA_2_ activity was associated with a 3-fold increased risk of death or recurrent myocardial infarction, independently of other established risk markers (41).

Several prospective epidemiological studies have reported that LP-PLA_2_ is a predictor of coronary artery disease, although controversy persists as to its independence from LDL-C. In the most recent, in coronary heart disease patients who were followed for 4 years, increased concentration of this phospholipase predicted future cardiovascular events (HR 2.65, 95%CI 1.47–4.76), independently of a variety of potential risk factors, including markers of inflammation, renal dysfunction and hemodynamic stress (42).

The NADPH oxidase constitutes the main source of superoxide in phagocytic and vascular cells. A recent study in a small series found increased circulating NADPH oxidase activity associated with carotid IMT, suggesting a relationship between phagocytic NADPH oxidative stress and the development of atherosclerosis (43).

### Proteolysis/fibrinolysis markers

#### Matrix metalloproteinases

MMPs are zinc-dependent endopeptidases with collagenase and/or gelatinase activity. Degradation of collagen fibrous cap may predispose atheromas to rupture. MMPs are highly expressed in atherosclerotic plaques, in particular in the shoulder regions. Whereas few data exist on the association between MMPs during ACS and cardiovascular outcomes, MMP-9 (gelatinase-B) levels are significantly increased in the coronary circulation in patients with acute MI and unstable angina (44). Circulating MMP-9 levels are also increased in type 2 diabetes patients with coronary artery disease, and elevated serum MMP-9 concentrations, associated with decreased inhibitor levels (TIMP-1), have been linked to premature coronary atherosclerosis (45–48). Recently, we demonstrated that MMP-10 (stromelysin-2) is associated with inflammation and subclinical atherosclerosis, and is also present in atherosclerotic lesions (27).

Pregnancy-associated plasma protein A (PAPP-A) was originally described as e peptide specifically elevated in pregnancy. Ruptured plaques have demonstrated PAPP-A expression in their shoulder. Some studies suggest that this peptidase may have diagnostic utility in identifying patients with ACS and without troponin elevation (49,50).

Despite some controversial reports regarding whether MMPs inhibition may have some deleterious effect, recent experimental studies demonstrate suppression of atherosclerotic plaque progression and instability by tissue inhibitors of MMPs (TIMPs), possibly through modulation of macrophage migration and apoptosis (51).

#### Fibrinolysis

Tissue plasminogen activator (t-PA) is a key component of the cardiovascular fibrinolytic system. In baseline conditions, t-PA is constitutively released from endothelial cells. Upon appropriate stimulation, substantial amounts of t-PA can be rapidly released resulting in a marked increase in fibrinolysis. The activity of t-PA in plasma is regulated by specific inhibitors. Of these, plasminogen activator inhibitor-1 (PAI-1) is considered to be the main inhibitor of t-PA in the vascular compartments, Additionally, α2-antiplasmin inhibits plasmin (the main proteolytic enzyme), thereby counteracting overhelming systemic fibrinolytic activity (52). There has been growing interest in the relationship of impaired fibrinolysis and coronary heart disease and stroke. Meta-analysis have shown that increased circulating levels of t-PA and PAI-1 are associated with cardiovascular risk. However, these associations were modest after adjustment for confounding established risk factors, and do not add significantly to the predictive value of current clinical risk scores (53).

### Biomarkers of platelet activation

CD40L, a member of the TNF family, is expressed by all the major cellular players in atherosclerosis, namely, activated T lymphocytes, EC, SMC, and macrophages. Studies examining circulating levels of soluble CD40L (sCD40L), which is primarily derived from activated platelets, have found elevated levels in patients with unstable angina and predicted the risk of future cardiovascular events in women. A recent study examining sCD40L in ACS found higher levels associated with increased risk of death or non-fatal MI, a risk that was significantly reduced with the glycoprotein IIb/IIIa inhibitor abciximab (54–56). However, a recent study failed to show an association between sCD40L and subclinical atherosclerosis (57).

### Genetic markers

The recent completion of the Human Genome Project has provided a great opportunity to identify high-risk patients through the use of technologies that integrate the entire genome. Assessment of genetic markers might predict risk of plaque instability or response to current therapies. For example, analysis of 112 polymorphisms in 2819 patients with ACS identified 3 genes (connexin 37, PAI-1 and stromelysin 1) associated with increased risk of MI (58). Another study examining 62 candidate genes in premature MI identified variants in 3 members of the thrombospondin gene family as risk markers (59). Current data also provide evidence for the role of MMP-3 polymorphism in plaque destabilization (60). Finally, polymorphisms in specific chemokine receptors were associated with serum MCP-1 levels and myocardial infarction in the Framingham study (61).

## Multi-marker Approach to Acute Coronary Syndromes

**Figure 1:**
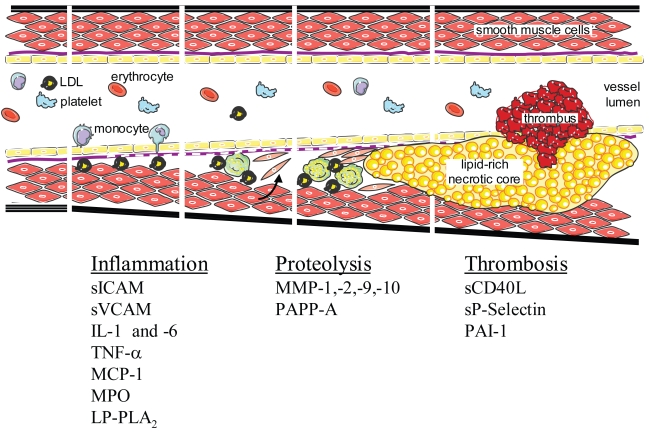
Multi-marker approach to atherothrombosis.

Advances in the understanding of the pathogenesis of acute coronary syndromes have stimulated development of novel biomarkers, and expanded their role in the different spectra of their underlying pathophysiology. This multi-marker strategy consists of an array of biomarkers assessing myocardial necrosis, plaque destabilization, myocardial stress, myocardial ischemia and inflammatory processes (62) (Figure 1). Those with potential clinical application include troponin T, for detection of minor myocardial damage associated with vulnerable plaque and thrombus, heart-type fatty acid binding protein (H-FABP) for earlier detection of myocardial damage (63), N-terminal pro-BNP for earlier risk stratification in cardiac emergency, and sCD40L for earlier identification of plaque destabilization with platelet activation (64).

## Integrating Soluble Biomarkers and Imaging Technologies in the Identification of the “vulnerable patient”: Bioimaging

Given the complex pathophysiology of cardiovascular disease, it is unlikely that any single biomarker will prove able to provide a universal surrogate of atherosclerosis. New imaging technology may be limited by technical difficulty, availability and cost. Soluble biomarkers may offer the advantage of availability and lower cost, but they may not prove as sensitive as imaging modalities in the detection or assessment of disease (65). Table 3 provides a list of molecules that may serve as candidates for non-invasively identification of high-risk atherosclerotic plaques in combination with imaging technologies (66, 67), It should be noticed, as shown in the IBIS trial (9), that weak correlations between circulating biomarkers and quantifiable imaging parameters are likely to be found.

In summary, with a multimarker approach combining emerging and new biomarkers with imaging technology, better risk profiles may emerge to provide prognostic information. A panel of inflammatory hemodynamic and vascular damage biomarkers, as part of a multimarker strategy (Figure 1), may help us to understand the pathophysiological mechanism underlying ACS and contribute to the development of new therapeutics in atherosclerosis (68,69).

Hopefully, bioimaging, by integrating biomarkers and imaging studies, offers a new opportunity to identify not only “vulnerable plaques” but also “vulnerable patients” for targeted therapeutic interventions.

## Figures and Tables

**Table 1:** Atherosclerosis imaging technologies.

Technique	Information	Advantages	Limitations
*Coronary angiography*	Coronary change score	Common procedure	Invasive
	Change in percent stenosis	Clinical experience	Provides lumen size only
*Carotid IMT*	Change in mean IMT	Noninvasive, availability and cost	Technically demanding Noncoronary assessment
*EBCT*	Change in Agatson score	Noninvasive	Limited reproducibility Not commonly used in clinical trials
*Brachial artery ultrasound*	Flow-mediated dilatation	Noninvasive, availability and cost	Need for standardized protocols
*IVUS*	Absolute change in plaque volume Percent change in plaque and atheroma volume	Direct imaging of the disease Standardized protocols Clinical experience	Invasive Plaque composition difficult to assess Assess anatomy, not function
*Magnetic resonance*	Change in mean vessel wall area Vessel calcification	Noninvasive Plaque characterization possible	No validation with clinical events
*OCT*	Unstable/vulnerable plaque Plaque composition	High resolution, high data acquisition rate Can be combined with adjuvant optical techniques	Invasive Attenuation by blood Limited penetration in tissue No validation with clinical events
*Virtual histology/IVUS*	Plaque morphology (lipid vs fibrous) Vulnerable plaque	High predictive accuracy Ability for detection of vulnerable plaques	Invasive Unable to differentiate thrombus No validation with clinical events

OCT: Optical coherence tomography; IVUS: Intravascular ultrasound; EBCT: electron beam computed tomography; IMT: intimamedia thickness.

**Table 2:** Bioimaging for identifying the vulnerable (high-risk) patient.

Arterial vulnerability	Blood vulnerability	Myocardial vulnerability
1) *Structural markers* - Carotid IMT - Coronary artery calcium	1) *Hypercoagulable* - Fibrinogen - D-dimer - Prothrombin fragment 1+2	1) *Structural markers* - LVH - LV dysfunction
2) *Functional markers* - Blood pressure - Endothelial dysfunction - Arterial stiffness - Ankle-brachial index - Urine albumin excretion	2) *Decreased fibrinolysis* - t-PA - PAI-1	2) *Functional markers* - Exercise stress test - PET
3) *Serological markers* - Abnormal lipid profile - Oxidized-LDL - LP-PLA_2_ - Inflammation - hs-CRP - Interleukins - SAA - MPO - sCD40L - Oxidative stress - Homocysteine - Natriuretic peptides - MMPS: -9, -10 - TIMPS	3) *Increased coagulation factors* - von Willebrand factor	3) *Serological markers* - Troponins - Pro-BNP - H-FABP

H-FABP: Heart-fatty acid binding protein; IMT: intimamedia thickness; LP-PlA_2_: lipoprotein-associated phospholipase A_2_; LV: left ventricle; LVH: LV hypertrophy; MMPs: metalloproteinases; MPO: myeloperoxidase; SAA: serum amyloid A; sCD40L: soluble CD40 ligand; PAI-1: Plasminogen activator inhibitor; PET: positron emission tomography; Pro-BNP: B-type natriuretic peptide; TIMPs: tissue inhibitors of MMPs; t-PA: tissue plasminogen activator.

**Table 3:** Panel of biomarkers potentially associated with vascular imaging.

Biomarker	Vascular imaging
Endothelial integrins (ICAM-1, VCAM-1, P-selectin, E-selectin) Ox-LDL MMP-2 and -9	MRI
VCAM-1, ICAM-1, E-selectin, CRP IL-6 LP-PlA_2_	IVUS
IL6, IL-10, Ox-LDL MCP-1 MMP-10 CRP Fibrinogen NADPH-oxidase	Carotid IMT
